# “You can be blind because of loving them so much”: the impact on owners in the United Kingdom of living with a dog with osteoarthritis

**DOI:** 10.1186/s12917-020-02404-5

**Published:** 2020-06-11

**Authors:** Zoe Belshaw, Rachel Dean, Lucy Asher

**Affiliations:** 1 PDSA Pet Hospital Nottingham, Dunkirk Road, Nottingham, NG7 2PH UK; 2 VetPartners, Leeman House, Station Business Park, Holgate Park Drive, York, YO26 4GB UK; 3 School of Natural and Environmental Sciences, Newcastle University, Room 608, Agriculture Building, Kings Gate, Newcastle, NE1 7RU UK

**Keywords:** Veterinary, Osteoarthritis, Canine, Burden of care, Impact, Human-animal bond, Welfare, Quality of life

## Abstract

**Background:**

There is growing awareness that caring for a chronically ill pet may have a detrimental impact on their owner’s quality of life. Companion animal orthopaedic disease has received little research interest in this context. Canine osteoarthritis is known to negatively affect the welfare of many dogs in the United Kingdom, but its consequences for their owners has not previously been described. The aim of this study was to use a qualitative methodology to explore the impacts on a dog owner that occur following their dog’s diagnosis with osteoarthritis. Owners of osteoarthritic dogs based in the United Kingdom (UK) were recruited through veterinary practices to participate in semi-structured interview about life with their dog. Interviews were transcribed verbatim and thematic analysis was used to construct key themes. This publication describes the theme that focused on the impact(s) that the dog’s condition had had on the life of their owner.

**Results:**

Forty owners of 35 dogs of a range of breeds and ages were interviewed. A wide range of negative impacts on the physical, mental and financial health of owners were described. Few had any prior experience of canine osteoarthritis, and owners of young dogs appeared to be particularly affected by the diagnosis. Owners detailed increasing worry over time about their pet’s condition, frequently combined with a growing need to physically assist their dog. Sometimes this led them to seek information about, and purchase, adjunctive therapies and products. The dog’s reduced mobility and need for medications progressively limited their own lifestyles and ability to have time away from their pet. Owners typically described a strong bond with their dog as a motivator to provide ongoing care.

**Conclusions:**

The negative impacts on owners of caring for an osteoarthritic dog appear multi-faceted and may be sustained over many years, particularly if the dog is young at diagnosis. Owners may be highly motivated to improve their dog’s mobility and to reduce the impact the condition has on their own lives, yet they may be unsure how to achieve this. Veterinary professionals should inform and support these owners as much as possible.

## Background

Dog ownership is increasingly advocated as part of a healthy lifestyle [1, 2]. Reported ownership benefits when compared to non-dog owners include: an increase in walking exercise independent of the weather [3]; increased chance of meeting recommended physical activity levels in children [4] and adults [5]; improved social contact and friendship building with others in the local neighbourhood [1, 6]; and more time spent in a natural outdoor environment [7]. Dog ownership may also bring disadvantages such as increased risk of injury from dog bites [8], challenges in finding and retaining rental accommodation [9] and the financial and emotional challenges associated with caring for a pet when it becomes ill, injured or dies [10–15].

Many dogs will suffer poor health during their lifespan [16]. Owners of dogs affected by chronic diseases including diabetes mellitus [17], epilepsy [18], heart failure [19], Chiari-like malformation [20] and cancer [21] describe significant, persistent worry about their pets’ condition. Those owners describe adapting multiple aspects of their own lifestyles to accommodate the reduced exercise capacity [15], requirements for time-specific medication dosing and frequent veterinary visits required by their dogs. In a series of recent cross-sectional studies, Spitznagel et al. [14, 22, 23] demonstrated, using scales designed for human patients and carers as well as for pet owners, that owners of pets with a range of chronic illnesses report greater “caregiver burden”, psychological distress and a lower quality of life than owners of healthy pets. Where research subjects, in this instance pet owners, are not involved in research design there is a risk that important topics may not be included in tools for their completion leading to poor face validity, a problem common also in human healthcare [24]. Qualitative research allows participants to describe their experiences in their own words which may identify previously undescribed, yet important, topics. Understanding these experiences can lead to important, generalisable insights for clinicians [25]. Little qualitative research has previously been performed in relation to ownership of chronically ill pets [11, 15, 26] and to date there has been scant research of any form exploring experiences of ownership of dogs with chronic orthopaedic disease.

Canine osteoarthritis is estimated to affect at least 2.5% of the veterinary-visiting dog population in the United Kingdom [27] and has recently been determined to have a high negative welfare impact when ranked against other common canine diseases [28]. It is a chronic, progressive disease [29] with some dogs affected from an early age [30]. Reductions in exercise ability and quality of life of osteoarthritic dogs are well documented [31–33], and owners may alter their own walking exercise and lifestyle to accommodate these changes [15]. However, the impact of canine osteoarthritis on the quality of life, emotional and physical health of their owners has not previously been explored in depth. This study therefore aims to answer the research question: what are the impacts on owners when their dog develops osteoarthritis? A qualitative methodology was used to ensure a wide breadth of in-depth experiences were captured.

## Results

Fifty-eight owners of osteoarthritic dogs expressed interest in participation. Fifteen subsequently declined to be interviewed, five were unavailable during the study period, four expressed interest only after the study had closed and two dogs were euthanased before the interviews with their owners could take place. Thirty-two interviews were conducted involving 40 participants who owned 35 osteoarthritic dogs of a wide range of ages, breeds and osteoarthritis locations and severities (see Supplementary Data 1 for full details). Only one dog had been acquired with pre-existing osteoarthritis; all others had developed the disease whilst under their current owners’ care. Male and female participants of a range of ages and backgrounds were recruited from the rural West Country to Glasgow city centre and fulfilled all aspects of the sampling frame (see Supplementary Data 2 for participant details). Interviews ranged from 52 to 170 min in duration, covering all aspects of life with the affected dog(s). From the complete dataset, themes were constructed from latent codes which captured underpinning ideas, and semantic codes that explained the surface meaning of the data. The following text summarises the theme describing the impact on owners of their dogs’ condition. It is accompanied by illustrative quotes from owners involved in the study.

### Finding out about the diagnosis

Few owners had any prior experience of managing a dog with osteoarthritis. Most were not particularly concerned about the impact the disease might have on their lives at the point of diagnosis. Many described their preconceptions of osteoarthritis as a progressive, incurable but negligible disease of older people and thought the same would be true in dogs, particularly if those dogs were older at the time of diagnosis. Amongst these owners, phrases such as “*I just thought …*” , “*I just assumed …*” , and “*Arthritis is just part of old age*” were commonly used. One owner described a fatalistic, almost hopeless, attitude to the diagnosis from the outset.

I just thought of arthritis as a progressive process. At some point that will in effect cripple her. [Interview 13]

Conversely, the diagnosis led to feelings of shock, sadness and guilt amongst owners of dogs that were young at the time of diagnosis. Consistently, these owners described thinking of their dog’s osteoarthritis as their own fault because they perceived it was not usual for a young dog to be affected. Several described trying to understand what they had done wrong with their dog’s nutrition, exercise or management that might have caused the problem. For some the diagnosis was associated with fear, bewilderment and a sense that the future would not be as they had envisaged it.

*Shock because it came out of the blue, he'd always been very sound. And because I hadn't heard an awful lot about elbow dysplasia within this breed, or any other come to that. And upset. Because I knew that's a lifelong condition, that's not going to get better. I'd got a dog who at four years old had got a condition that was going to gradually get worse, and his lameness would get worse.* [Interview 5]

*When they said “Yeah, no, it is arthritis” I felt out of control, because I couldn't do anything about that because she had it. You can't chisel it away and start again, it is there. And, I kind of knew it's only going to get worse, because it does in any walk of life. And that was probably my limit of what I knew. And that scared me. She was, what, two-and-a-half, three, so she was young.* [Interview 20]

*I remember coming home crying, I just absolutely, was on the phone to Mum, and I thought it was the end of the world. I thought 'She's a young puppy still, I don't know how I'm going to deal with this. And she's got arthritis in both hips.'* [Interview 21]

*When we got the diagnosis, and we're there thinking 'What have we done wrong? Is it something we've done?' and [husband’s name] said “Well, do you think it's something to do with her being on a hard floor, from being a puppy … Has that destabilised her hips? Maybe it's something like that?”* [Interview 23]

Owners who had previously managed a dog with osteoarthritis described the benefit of this experience. An owner of two dogs included in the quotes above described the difference in attitude to the diagnosis of osteoarthritis in her second dog compared to her first, demonstrating her increased confidence and empowerment.

*I wasn't as frightened about the condition as I was then. Because I thought it was the end of the world when [dog 21A] got diagnosed, whereas with [dog 21B], I was like 'Okay, I know how to deal with it.'* [Interview 21]

*With Shepherds it's something that you would think 'This is likely to happen', so no surprise. I'd actually already started meloxicam [before we went to the vets], because we had it here, and I think would normally be one of the first line treatments anyway … .* [Interview 4]

### The multifaceted negative impacts of owning an osteoarthritic dog

Every interviewee described a range of impacts on their own daily lives following the diagnosis of osteoarthritis in their dog(s). Many related a heightened sense of worry and concern. Causes included: the stress of veterinary visits; feeling they had let their dog down by failing to find effective treatments; challenges of getting the dog to take medications; and keeping the dog at a reasonable weight and body condition score whilst on a restricted exercise regime. Many affected dogs were described as stiff, slow, reluctant or unable to walk long distances, less willing to play, less able to navigate steps and stairs, and to spend more time resting. All had become more reliant on their owners to help them physically, and sometimes emotionally; the disease was described by some to be accompanied by a marked loss of confidence in the dog. Frequently, owners struggled to understand what these different behavioural changes meant, particularly in older dogs. For most, the dog’s osteoarthritis was a dominant presence in their home lives.

*I was always looking for her to come back to the point where it had started. Could never get into my head that actually it was never going to be like that.* [Interview 15]

*You have to look after them, you have to make sure that they don't hurt themselves, for instance that they don't do silly things. You have to actively look for signs as well which tell you how they feel, whether they've got a good day or a bad day, whether they're more painful or less painful. And that does become part of your daily routine, and was never really part of your daily routine when they were young because they were healthy, playful, up for anything. And you didn't really have to worry. And now you're much more worried about them* [Interview 19]

Most dogs had experienced some initial benefit from veterinary prescription treatments such as non-steroidal anti-inflammatory drugs, but almost all owners described an apparent reduction of efficacy with time as disease severity increased. Several owners described a continual search for a treatment that would return their dog “back to normal”. Some felt frustrated that their veterinary surgeon had not provided them with good information about the availability, or likely efficacy, of additional therapies or non-prescription medications, though others had had an excellent experience. If their veterinary surgeon did not provide the information they sought, some owners joined internet forums or followed up recommendations from other owners they encountered on dog walks.

*From experience, vets aren't always as up-to-date as maybe they could be. I want to be tactful about it. Our vets are very good, but you know as well as I do that they don't all keep as up-to-date. And some of them have favourite things that they've always done because they've always done it.* [Interview 25]

*I said to the vet that we'd been going to just recently, I said “Can she have a physio assessment?” and she said “That's a really good idea, yes, that would...” And I thought 'Why didn't you say before?' instead of just giving us the painkiller?* [Interview 7]

*And the butcher’s daughter sees me walking around town. And one day she said “Your boy's limping.” And I said “Yeah, he's got arthritis.” She said “Devil's claw.” And I said “Well what in heavens is that?” And she told us, she said “It's quite expensive...”, and it is quite expensive. It's twelve ninety-nine for fifty tablets. [ … ] Still cheaper than meloxicam. But we said “Oh, we'll give it a try, it can't do no harm.” So, that's how we got on it, never heard tell of it before.* [Interview 24]

Owners had invested in car ramps, orthopaedic dog beds and a huge range of other non-prescription therapies, remedies and products. Frequently, these products were found not to be as effective or well designed as they had hoped. Sometimes owners were unsure of the impact that the adjunctive treatment or product was making, but were reluctant to stop in just in case it was doing something useful. For some owners, the disease became financially significant due to the cost of medications, adjunctive treatments and veterinary visits. A couple had left their jobs as a direct result of their dog’s osteoarthritis; others had changed work schedules, room layouts, vehicles and furniture.

*He won't walk up a ramp. My friend's got one, and she brought it round, and even with my husband one side, me on the other, and a bowl of food in the car, he wasn't going up that ramp.* [Interview 5]

*You see, her [magnetic] collar made a big difference to her at first, and she wears it most of the time. And I think 'Well, is it still giving her the same benefit as it did at first?’* [Interview 7]

*Going back to when she was two, it was literally, right, so I immerse myself in as many textbooks as I could, I became a canine hydrotherapist, and we did acupuncture, we did chiropracty. My friend’s a physio, so she did laser treatment with her. Gosh, you name it basically.* [Interview 21]

Owners also described direct negative consequences to their own physical health as a result of their dog’s disease. Several had developed back pain from carrying or supporting big dogs up and down stairs or helping them in and out of the car. At the time of the interviews, several dogs walked only a few hundred metres each day with one dog not even leaving the garden. Others were estimated to be covering several miles on a good day. In addition, owners who had had walked their dog several miles to be cared for by a friend or relative whilst they were at work were now having to deliver the dog by car or public transport. Some owners, both male and female, described their guilt at going for a walk alone, leading them to significantly reduce their own exercise rather than leave their dog behind. Social isolation, boredom and reduced personal fitness were direct consequences for some. Owners of young dogs who had looked forward to years of activity together appeared particularly affected by the change in their dogs’ exercise ability.

*So yeah the car thing is definitely the thing that at the moment is probably the biggest challenge. Because I've got a bad back, so I shouldn't really be lifting a thirty kilogram dog in and out of the car. But I don't really have a lot of choice...* [Interview 1]

*I'd walk her maybe three miles to my mum's and leave her there and then pick her up when I came from work, and would maybe walk home again. So she became much less portable. Because what we found was things like sitting in the car, that squinched whatever, and if she sat in the car she couldnae walk when she got out, she'd be all staggery in the back legs and things so obviously she couldnae go in the car as much, I couldnae take her on the bus, so I’m very limited now. But what we do is, the train station's five minutes over there, and my mum stays literally two minutes from the train station, so she can still get down to my mum's. I take her on the train.* [Interview 27]

*So I'm not getting as much exercise. And I don't like going out with my daughter on my own without him. Because he knows I'm going out for a walk. And his little face looking out of the window when you're going. That's how it's affected me, yeah. It's sad really.* [Interview 2]

*When we go to Northumberland she would run along the beach, walk up the hills. So we envisaged that we would be doing that a lot more with her, particularly as our two children got older, and when we go walking, take her with us. And now we think actually, probably not …* [Interview 23]

Respite from caring was difficult to achieve since many owners no longer felt comfortable leaving their dog in kennels or with a friend. Some were worried about their dog’s needs not being met whilst others felt it was unfair to burden someone else with the requirements of their osteoarthritic dog. Several owners, including one with young children, had not had a holiday away from home for several years because of their dog’s disease. A few dogs had taken part in showing or agility before their diagnosis. Much of their owners’ free time had been spent enjoying these activities with their dog, often travelling widely and being part of a community who shared the same interest. These owners described how the diagnosis of osteoarthritis in their dog had, or threatened to, remove them from this community.

*She's too old to go in a camper van, because that was our holiday, there was a camper van into France and Spain and Austria, places like that. And that was very good. And now, I can't take her, I won't put her into kennels, I can't take her in the car, so it's a severe restraint on me. I just can't take a holiday. Well, I won't take a holiday for as long as I've got her. I'm stacking them up now, all the holidays, cruises here, and Peru and things like that. And I've started a big file upstairs on different holiday destinations.* [Interview 13]

Female owner: *But it was a big blow to you, wasn't it, to lose your agility. Because that's really the only hobby [he] has outside the house. Whereas I've got other hobbies and other interests. So I get out for other things. But it's when your social interaction and things like that, you lose that. And that's a bit of a shame and a bit of a shock, isn't it*. Male owner: *That's right, it is, yeah.* [Interview 8]

*I have to be honest and say I didn't really ask an awful lot in the breed at that time* [of the diagnosis]*, because I was still hoping to show him. And if you disclose at that stage, this is where it's difficult, if you disclose that your dog's got this sort of issue, then no matter how soundly he goes round the ring, people will see him limping.* [Interview 5]

Decisions about when their dog would need to be euthanized weighed heavy on the minds of most owners, even those of dogs that were not yet badly affected. Most described having very little idea about how they would make a decision with some hoping that their vet would provide guidance as needed. Whilst some felt that their veterinary surgeon would be able to help them make the right decision at the right time, others felt isolated in their decision making. Several expressed a desire for greater availability of information about the condition and its impacts, and better support from their veterinary team.

*And I think that if she had some other medical condition that you knew, well, she's in too much pain, or she can't deal with this, 'Right, this is the end.' It's much easier than something like arthritis that's slippery slope. And, well, how do we know? We don't know.* [Interview 15]

*The only thing I hope is that when I wake up one morning … she passes when asleep. I don't want to make a decision. That's the only thing. I don't want to do that, but if I have to do it, I have to do it. It's a family thing, but obviously I'm the main one that's got to make a decision.* [Interview 28]

*You can be blind because of loving them so much, so I think you need a bit of help when things get really hard.* [Interview 16]

### Looking for the positives

A striking aspect of these interviews was the ongoing love and devotion owners continued to have for their dogs despite their changed needs and shifted relationships. At the outset of interviews, most dogs were described as “*just a pet*” but the relationship between owner and dog in almost all instances was clearly much more complex. Owners of a range of ages and backgrounds described how their dog continued to help them emotionally in times of stress and crisis, through new jobs, house moves, bereavements and divorces by providing continuity and unconditional love. Additional bonds of varying strength and significance were described between each dog and husbands, wives, grandparents, friends, children or local dog walkers. Some relationships with others were sporadic or transient, others constant, but all were perceived by the dog’s owner to be important both to dog and person. In return, dogs’ needs were met, their behavioural quirks accommodated, and stolen or chewed objects forgiven.

*He is my companion, that's basically it. So he comes with me wherever I go, he comes with me even into work. And yeah, everything we do together … .So, he was more than that to be honest, because, yeah, I was going through some tough periods of life, he was always there. So he was a big mate, a companion, a soul mate.* [Interview 19]

*So she was bought as a family pet and that's just what she is, she's not got any other great function other than … she's actually been great for me. I'm a firefighter and my work’s quite stressful at times and I find that she's very really a stress reliever just going out for a walk and just getting out of the house in all sorts of weathers.* [Interview 30]

Several owners compared a shift in their relationship from that of mutual dependence to now being like a carer for an elderly relative or close friend. Almost all were clear to emphasise that whilst they were sad about their dog’s osteoarthritis, they had no resentment for the impacts they experienced as a consequence of their dog’s disease because their relationship with their dog continued to be a hugely important part of their lives.

*Again, because you've built that relationship, you've had that fun together. If it was the other way... If you like, if I was blind I'd probably have a dog guiding me. So, if she's getting to the point of having a few difficulties I'm going to care for her, yeah.* [Interview 11]

*We just all love her, and she's just like your old granny or something, she's just an old lady. We just look after her and make allowances for what she can do and what she can't do.* [Interview 27]

## Discussion

This qualitative research involving 40 dog owners is the first to identify that that many aspects of owners’ lives may be negatively affected when a dog becomes osteoarthritic. Given the prevalence of osteoarthritis in the canine population [27] and the estimated frequency of dog ownership in the United Kingdom [34], millions of people could be facing previously unreported adverse impacts on their health and wellbeing as a consequence of this disease. Our findings suggest that canine osteoarthritis may diminish many of the positive health benefits associated with dog ownership, and this should be considered when managing a dog with this condition. Importantly, these interviews suggest that canine osteoarthritis does not appear to disrupt the attachment many owners have to their dogs, with some owners describing the disease to have brought them closer to their pet. This appears to be a strong motivator both to care for them and to seek solutions to improve their comfort, and this attachment may lessen the perceived impact of the lifestyle changes experienced.

These interviews add to a growing body of literature describing objective and subjective impacts experienced by owners caring for ill animals. These results describing multifaceted negative impacts on owners’ lives add to the recently published data describing the impacts on dog walking activity when the dog develops osteoarthritis [15] and reflect those of other studies looking at the impact of a range of non-orthopaedic diseases in pets. Depression, anxiety, stress and a sense of a reduced quality of life were also found to be prevalent in American owners of ill pets [14], whilst social isolation, guilt, worry and responsibility were described by Danish owners of ill pets interviewed by Christiansen [11]. Negative impacts on the lifestyle of owners were considered by 32% of respondent veterinary surgeons to be a cause of euthanasia in dogs with diabetes mellitus [35]. Freeman [36] described similar physical labour associated with caring for physically incapacitated dogs with severe spinal cord disease. Canine osteoarthritis should therefore be added to the list of diseases that may have substantial negative consequences for the lives of owners. Future studies might consider comparing similarities and differences between negative impacts created by different conditions, and further explore the link between ill pets and human wellbeing.

In human healthcare, patient and carer support groups are commonplace, funded by pharmaceutical companies, charities and governments. At the time these interviews were conducted, little external support was available for owners of dogs with osteoarthritis. Traditionally, support for owners would be considered to be provided by the veterinary practice with the veterinary surgeon acting as diagnositician, key information source and gateway to accessing prescription medication. However, Christiansen and colleagues [26] identified that owners may not receive the support that they seek from their veterinary practice in relation to end of life decision making, and Belshaw and colleagues [37] identified mismatched expectations between veterinary surgeons and owners during preventive healthcare consultations. This is further illustrated by the anonymous owner of an osteoarthritic dog who wrote compellingly about her sense of isolation when she felt her local practice was not giving her the support she needed [38]. Recently, owner-to-owner support networks have developed via social media, with owners of dogs with epilepsy describing online forums as an important source of support [39]. Since these interviews were conducted, a free online owner support network has been established for owners of osteoarthritic dogs. “Canine Arthritis Management” [40] now has many thousand followers on social media, and whilst its impact has yet to be objectively determined, its popularity suggests it is meeting an important need. Access to good quality information and support should facilitate better decisions to be made, and may help safeguard owners’ own wellbeing, enabling them to continue care provision [41].

Despite significant impacts on their own lives, few owners appeared to resent their change in circumstances. Many articulated their strong, and in some instances increased, attachment to their dog as an explanation for their perseverance. Previous research has illustrated the presence, and importance, of attachment and emotional relationships between pet and owner, which may vary with age and health state [42, 43]. Using a strange situation test, Mongillo and colleagues [44] identified that a cohort of older dogs appeared more attached to their owners than younger dogs, and suggested that these dogs may have increased reliance on their owners for emotional support. This was affirmed by owners in the current study, though it is possible that both studies selected for those owners who felt that the bond had been altered. Most owners of dogs with severe spinal cord injuries that required significant nursing care also described an increased bond with their dogs as a result of caring for them [36]. However, strong attachments between owner and pet have been associated with delayed euthanasia decisions leading to welfare compromise [45]. Owners in this study and others [11, 18, 26] described struggling to recognise chronic pain, assess quality of life and identify the right time to euthanase their dog. These results suggest that practical quality of life assessment tools may be beneficial to owners of osteoarthritic dogs [46] and that veterinary surgeons may need to increase their support and guidance for owners considering end of life decisions.

The rich narratives collected as a result of this research demonstrate the advantage of using a qualitative methodology in exploring the actions and the motivations of pet owners. Whilst this method involved fewer participants than would have been included in a quantitative study, it allowed us to describe aspects of the life of a pet carer which may otherwise have been missed. Our interviews captured experiences of owners of a range of ages, socio-economic situations and locations within England and Scotland. Unfortunately, funding did not permit inclusion of owners from a wider range of locations. The majority of interviewees were female. This is compatible with other UK-based companion animal research involving owners [37] and is reflects a finding of greater levels of dog ownership amongst female study participants [47]. Additionally, women are more likely than men to take on caring roles in the household [48] and future research should investigate whether women also typically take on the role of pet carer in UK households. Some dogs were included in this study that did not have a definitive, radiographic diagnosis of canine osteoarthritis. Focus groups with general practitioners [49] suggested that diagnosis of canine osteoarthritis in a general practice setting is often based on clinical history, physical examination and response to treatment rather than radiography. We therefore considered that insistence on a definitive diagnosis would exclude a large number of dogs from our target population, and that a definitive diagnosis was unlikely to have a significant effect on the impact of the disease on owners. Dogs with comorbidities were included since definitively excluding any comorbidity in this predominantly older dog population was impractical, and our aim was to include the widest possible range of affected dogs. Interviews concentrated specifically on the impact of the dogs’ osteoarthritis on their owners so we do not consider this to be a significant limitation. Whilst these data are now several years old, this remains the first research of its kind in this disease. This study was limited to a relatively small number of owners and should not be seen to be representative of the experiences of all dog owners, but these data provide an excellent framework from which further quantitative research can be performed to determine how widespread these experiences are in the UK and beyond.

## Conclusions

The impacts on owners of caring for osteoarthritic dogs appear multi-faceted and may be sustained over many years. As such, the human health benefits associated with owning a dog may be diminished when that dog develops osteoarthritis. Despite the apparent prevalence of this disease, dog owners did not appear particularly aware of the condition or the impact it might have on their lives, and access to good quality information about the management options appeared poorly available at the time of these interviews. Owners of affected dogs need both information and support to help them optimally manage their dogs’ condition. Veterinary surgeons therefore have an important role to play in improving the welfare of both osteoarthritic dogs and their owners.

## Methods

Data presented are from drawn from a detailed qualitative study, conducted at the School of Veterinary Medicine and Science, University of Nottingham, which used interviews and focus groups to explore the experiences of dog owners, veterinary surgeons and veterinary nurses who manage dogs with osteoarthritis [49]. Some results and methodological details have previously been reported [10, 15]. Reporting follows the Consolidated Criteria for Reporting Qualitative Research (COREQ [50];).

### Interview process

The inclusion criteria for interviewees were: a) ownership of a dog at least 5 years of age treated or managed for osteoarthritis in at least one limb following a diagnosis of osteoarthritis by a veterinary surgeon; AND b) residency of dog and owner(s) in the UK. A diagnosis of osteoarthritis by radiography was not necessary for inclusion. Dogs with known comorbidities were not excluded, and examination of the dogs to look for undiagnosed comorbidities was not performed.

Recruitment was based on a purposive sampling frame constructed by the authors (see Supplementary Data 3) designed to capture the widest possible range of UK owner experiences (eg including a range of owner ages, geographic locations and dog breeds). Interviewees were recruited through: displaying information posters in a convenience sample of 10 veterinary practices in England and Scotland asking owners of osteoarthritic dogs to contact the lead author; snowball sampling where recruited owners find other eligible owners; and from the authors’ personal networks. Incentives to participate were not offered. Interested owners were sent information about the purpose of the study including details of the interviewer (ZB)'s background as a veterinary surgeon and previous owner of an osteoarthritic dog. If they were willing and eligible to participate, an interview date was arranged. All interviews were conducted face-to-face by ZB in owners’ homes between February and August 2014. A semi-structured interview guide, piloted with eligible owners before use (see Supplementary Data 4) covered eight broad topics ranging from the owners’ acquisition of, and relationship with, their dog to the way treatment decisions were made. The guide included initial questions on each topic with multiple follow up questions. This was used as a prompt to ensure important areas were not missed but owners were encouraged to lead the interview. All family members who had a direct role in caring for an eligible dog were invited to participate, and all eligible dogs within each household were discussed. Pertinent to this publication, interviews explored how owners’ lives had been changed since their dog had been diagnosed with osteoarthritis.

### Thematic analysis

Interviews were audio recorded using a Dictaphone and were professionally transcribed intelligent verbatim. Transcribed interviews were reviewed several times by the lead author in combination with contextual field notes made during the interviews. They were checked for accuracy against the audio recording but were not returned to interviewees. Thematic analysis was performed by ZB with assistance from the other authors following the six step plan described by Braun and Clarke [51] using the organisational support of nVivo (nVivo v10, QSR). Interview data were categorised line by line into latent and semantic codes from which subthemes and themes were actively constructed. Analysis was performed in parallel with data collection; constant comparison was used to ensure all interviews, and therefore the full range of opinions and experiences, were included [52, 53]. Statistical analysis was not performed as the qualitative purposive sampling methodology aimed to capture a wide range of experiences rather than to represent a population [53, 54]. The number of participants included was considered sufficient for this exploratory research given the tight inclusion criteria, in-depth nature of the interviews, the personal experience of the interviewer in the situation discussed and the cross-case analysis performed [55].

## Supplementary information

**Additional file 1 Supplementary Data 1.** Table describing dogs discussed during the interviews as recalled by their owners. Detailed table describing: age; breed; sex; age at diagnosis; comorbidities; limb(s) affected; joint(s) affected; diagnostic tests performed; and treatments trialled of dogs included in the study as described by their owners. **Supplementary Data 2.** Table summarising the interview participants’ coverage of the sampling frame. Enumerated summary of the participant and household data of interviewees related to the purposive sampling frame constructed. **Supplementary Data 3.** Purposive sampling frame used to recruit dog owners to the study. Complete purposive sampling frame used to recruit owners for the study. **Supplementary Data 4.** Interview guide used during semi-structured interviews. Complete interview guide used to collect data presented in this study.

## Data Availability

The datasets generated and/or analysed during the current study are not publicly available as they contain information that could compromise participant privacy and consent, but are available from the corresponding author on reasonable request.

## Abbreviations

COREQ: Consolidated Criteria for Reporting Qualitative Research
UK: United Kingdom
