# Supplementary material for: “You can be blind because of loving them so much”: the impact on owners in the United Kingdom of living with a dog with osteoarthritis
Source: BMC Vet Res. 2020 Jun 11;16:190. doi: 10.1186/s12917-020-02404-5 (PMC7291569; doi:10.1186/s12917-020-02404-5)
Supplement: Supplementary file 1 — Additional file 1 Supplementary Data 1. Table describing dogs discussed during the interviews as recalled by their owners. Detailed table describing: age; breed; sex; age at diagnosis; comorbidities; limb(s) affected; joint(s) affected; diagnostic tests performed; and treatments trialled of dogs included in the study as described by their owners. Supplementary Data 2. Table summarising the interview participants’ coverage of the sampling frame. Enumerated summary of the participant and household data of interviewees related to the purposive sampling frame constructed. Supplementary Data 3. Purposive sampling frame used to recruit dog owners to the study. Complete purposive sampling frame used to recruit owners for the study. Supplementary Data 4. Interview guide used during semi-structured interviews. Complete interview guide used to collect data presented in this study. [file 12917_2020_2404_MOESM1_ESM.docx]

**Supplementary materials, Additional File 1**

**Supplementary Data 1. Table describing dogs discussed during the interviews as recalled by their owners**

| **Interview number** | **Dog ID** | **Age (Years)** | **Breed** | **Sex** | **Comorbidities of which owners were aware** | **Age at diagnosis** | **Limbs affected** | **Joint(s) affected (if known)** | **Diagnosed by radiography or other advanced imaging?** | **Known underlying cause of osteoarthritis in any site?** | **Current treatment for osteoarthritis (as listed by owner)** |
| --- | --- | --- | --- | --- | --- | --- | --- | --- | --- | --- | --- |
| 1 | 1 | 14 | Labrador | M | Dilated cardiomyopathy, glaucoma, deafness, food intolerance, laryngeal paralysis, early chronic kidney disease | 9-10y | LH, RH | Hips | Yes | Hip dysplasia | Tramadol |
| 2 | 2 | 10 | German Shepherd cross | M | Behavioural problems | 10.5y | LH, RH | Hips | No | No | Meloxicam, Joint supplement |
| 3 | 3 | 10 | Staffordshire Bull Terrier | F | No | 11 months | LF, RF, RH | Stifle, elbows | Yes | Cruciate | Fish oil, green lipped mussel, serapeptase, hydrotherapy, physiotherapy, tramadol after hydrotherapy, laser therapy |
| 4 | 4 | 7-10* | German Shepherd cross | M | No | 6-9y | All limbs | Elbows confirmed | Yes, confirmed elbows and excluded hip dysplasia | Bilateral limb deformity | Meloxicam, gabapentin |
| 5 | 5 | 7 | Spinone | M | Colitis | 4 y | LF, RF | Elbows | Yes | Elbow dysplasia | Robenacoxib, Pentosan polysulfate, magnetic collar |
| 6 | 6 | 12 | Collie | M | No | 6y | LH, RH | Hips, stifles | No | No | Pentosan polysulfate, robenacoxib as needed |
| 7 | 7 | 10 | Labrador | F | Liver disease | 6y | All limbs | Shoulder, elbow, hips | Yes for elbows and shoulders | No | Cimicoxib, joint supplement, hydrotherapy, magnetic collar |
| 8 | 8A | 8 | Miniature Poodle | M | No | 8y | LF, RF | Carpi | No | No | Pentosan polysulfate |
| 8 | 8B | 11 | Miniature Poodle | F | No | 11y | LH, RH | Hips | No | No | Pentosan polysulfate |
| 9 | 9A | 14 | Basset | M | No | 8-9y | LF, RF | Carpi | No | No | Hills j/d food, firocoxib |
| 9 | 9B | 8 | Springer Spaniel | F | Behavioural problems | 4 months | RH, LH, | Elbow, hips | Yes both sites | Fracture | Hills j/d diet, firocoxib, Pentosan polysulfate |
| 10 | 10 | 11 | Labrador | F | Laryngeal problem | 9y | RH, LH | Hips. stifles | No | No | Carprofen, joint supplement |
| 11 | 11 | 14 | Labrador | F | Separation anxiety, deafness, intermittent faecal incontinence | 2y | LF | Carpus, feet | No | Limb deformity following fracture | Firocoxib, intermittent tramadol, joint supplement, cod liver oil |
| 12 | 12 | 10 | Australian terrier | M | No | 7-8y | LH, RH | Stifles | No | Cruciate repair both stifles | Carprofen, joint supplement |
| 13 | 13 | 14 | Giant Schnauzer | F | Urinary incontinence, deafness, hypothyroidism, laryngeal paralysis | 11y | RH | Hip | No | No | Carprofen |
| 14 | 14 | 9* | Large crossbreed | M | No | 7y | RH, LH | Stifle | Yes | Cruciate repair both stifles | Robenacoxib, tramadol |
| 15 | 15 | 11 | Labrador | F | Deafness, laryngeal paralysis | 8y | All limbs | Shoulder worse than others | Yes | No | Firocoxib tramadol as needed, ranitidine |
| 16 | 16 | 10 | Rhodesian Ridgeback | F | Amputated LF | 8y | RF, back | Unknown | No | Likely from forelimb amputation when puppy | Carprofen |
| 17 | 17 | 17 | Shih Tzu | F | Deafness | 15y | LF, RF | Unknown | No | No | Joint supplement, robenacoxib, pushed in cart on long walks |
| 18 | 18 | 13 | Springer Spaniel | M | Deafness | 7y | LH | Hip | Yes | Dislocated hip | Carprofen as needed, Nutroquin, |
| 19 | 19 | >13* | Large crossbreed | M | No | 8y | RF, LH, RH | Carpus, hips | No | No | Amantadine, gabapentin |
| 20 | 20 | 10 | Labrador | M | Multi-focal lumbosacral disc disease | 3y | All limbs | Hips plus others | Yes (hips) | Hip dysplasia, elbow dysplasia | Gabapentin, firocoxib |
| 21 | 21A | 9 | Labrador | F | No | 2y | All limbs | Hips plus others | Yes (hips) | Hip dysplasia | Tramadol, gabapentin, laser therapy [joint replacement] |
| 21 | 21B | 8 | Bassett | F | Chronic obstructive pulmonary disease, urinary incontinence | Unknown | All limbs | Right stifle and carpus most significant | Yes | No | Joint supplement,  acupuncture, massage, tramadol as needed |
| 22 | 22 | >12* | German Shepherd cross | M | Urinary incontinence, deafness, poor eyesight, laryngeal problem | 9y | All limbs | Unknown | No | No | Firocoxib, tramadol |
| 23 | 23 | 6 | Labrador | F | No | 3y | LH, RH | Hips | Yes | Hip dysplasia | Joint supplement, orthopaedic bed |
| 24 | 24 | 10 | Border Collie | M | No | 8y | LF | Elbow | No | No | Devil’s Claw, joint supplement |
| 25 | 25 | 13 | Labrador | M | Deafness | 2y | LF, RH, LH | Shoulder hips | Yes | No | Metacam, joint supplement |
| 26 | 26 | 9 | Boxer | F | Urinary incontinence | 3y | LH, RH | Stifle, hock | No | Cruciate and achilles tendon ruptures | Meloxicam, tramadol |
| 27 | 27 | 13 | German Shorthaired Pointer | F | Deafness, suspected nasal mass | 11y | LH, RH, (one FL) | Hips | No | No | Meloxicam, tramadol, joint supplement |
| 28 | 28 | 17 | Border Collie | F | Hyperadrenocorticism cognitive dysfunction, deafness, poor eyesight | 15y | LH, RH, LF | Hips, | No | No | None |
| 29 | 29 | 11 | Small crossbreed | M | No | 9y | LF, RF | Elbow | Yes | Angular limb deformity | Paracetamol |
| 30 | 30 | 9 | Soft Coated Wheaten | F | No | 6y | RH | Stifle | Yes | Cruciate repair | Firocoxib, seroquin, ranitidine |
| 31 | 31 | 9 | Labrador | M | No | 8y | LH RH | Stifle, hip | Yes | Cruciate repair | Firocoxib, joint supplement, tramadol |
| 32 | 32 | 9 | Greyhound | M | Deafness | 8y | LF, RH | Hip, carpus | No | Speculated to be injured during racing | Joint supplement |

Legend: M – Male; F – Female; y – years; LF – left fore leg; RF – right fore leg; LH left hind leg; RH – right hind leg; EFA – essential fatty acid

* Rescue dog, age estimated.

**Supplementary Data 2. Table summarising the interview participants’ coverage of the sampling frame**

|  | **Category** | **Sub categories** | **Frequency** |
| --- | --- | --- | --- |
| **Owner factors**  **(n=40)** | Sex | Male | 11 |
|  |  | Female | 29 |
|  | Age (as judged by interviewer) | 18-35 years | 2 |
|  |  | 36-65 years | 24 |
|  |  | 66 years and over | 13 |
|  | Ethnic minority | Yes | 1 |
|  |  | No | 39 |
|  | Previous dog owning experience as adult | Yes | 30 |
|  |  | No | 10 |
|  | Experience of owning a previous arthritic dog | Yes | 12 |
|  |  | No | 28 |
| **Household factors (n=32)** | Housing status (may cover more than one category) | Single | 5 |
|  |  | Cohabiting couple | 27 |
|  |  | Elderly parent living in home | 2 |
|  |  | Children under 18 living in home | 8 |
|  | House access type | Difficult access e.g. flight of stairs to enter | 1 |
|  |  | Other | 31 |
|  | House area | Rural | 10 |
|  |  | Urban | 12 |
|  |  | Conurbation | 10 |
|  | More than one dog currently living in house | Yes | 11 |
|  |  | No | 21 |

**Supplementary Data 3.** **Purposive sampling frame used to recruit dog owners to the study**

Sex: male; female

Ethnic minority? Yes; no.

Age: 18-35; 36-64; 65+

Previous dog ownership as an adult? Yes; no

Household type: Single; cohabiting couple; others living in household

Home area: Rural; urban; conurbation

House access: Easy for dog to access; difficult, e.g. Flat/apartment upstairs

**Supplementary Data 4.** **Interview guide used during semi-structured interviews**

1. Tell me about [your dog] first of all in terms of a general background, how long you have had them for, any health problems he/she has other than OA etc?

a. How old was he/she when acquired and where did they come from?

b. Concurrent diseases

c. Previous dog owning history? Other dogs now? Any with OA?

d. Use of dog – working, pet etc – relationship with/purpose of that dog/ special bond with this one?

e. Dog role in the house

f. Any human experience with arthritis? Any with other pets?

g. Any kind of dog training philosophy – importance of that?

2. We’re going to talk about the diagnosis now and your information sources. What signs did you first notice which made you think [your dog] might have arthritis, and what happened from there?

a. When was it that the dog got OA? Was it easy to define a moment or really insidious?

b. What clinical signs were there which made you think it was likely?

c. Why did you decide to go to the vet?

d. How did they diagnose OA?

e. How did you feel about the diagnosis? Surprise? Did you expect it – if so, since when? Did that help?

f. What about the prospect of starting on treatment – how did you think about that?

g. What information did they give you about the disease? Was it useful?

h. What did you know about OA before that? Where from?

i. Did you receive any information about how to manage your dog at home from the vet? Was it useful?

j. Which information sources have you used since – friends, internet, books etc if any – importance of breed specificity in that?

3. Now we’ll move on to talking about treatments and monitoring. So [dog name] is on some treatment for the arthritis. Tell me about the treatments [your dog] is on – how you decided on them, how well they work, any problems or worries about them etc. Treatments include any supplements, physiotherapy, magnetic collars, swimming, acupuncture etc as well as drugs.

a. How did you come to a decision about how to treat [your dog] with the regime you started with?

b. What treatments have you used, has it changed over time, why did you chose it (diet, nutra, drugs, supplements, physio etc) TIME FRAME?

c. What were your hopes for that treatment in terms of how it would change things?

d. How do you feel about your dog being on that treatment? Peace of mind that in less pain?

e. Did or do you have any concerns about side effects? Positives and negatives about treatment? What have you done about those concerns – advice sources etc? Information on s/es adequate?

f. Do you use the medication as advised or do you sometimes change the schedule? Do you do anything else other than what your vet has advised which you think helps? Do you find it easy to follow what was advised or do you sometimes forget doses etc – others say they do! Have you had any problems with administering any treatment?

g. Have you added any supplements or other therapies which your vet didn’t tell you about e.g. magnetic collar, massage, joint supplements?

h. Do you monitor your dog’s arthritis at home – if so, how? Any advice from vets/others on monitoring at home?

i. How do you tell whether or not a medication is working?

j. How to tell a good day from bad one – best tests? How easy is it to tell that the dog is having a good versus bad day - stoic personalities or easy to tell – how?

k. Do you need to go for re-check s to your vets? Do you see a vet or a nurse? What do they do? Do you find them useful? Why? How could the visit be made more useful?

l. Support with the disease – from others, vets, dog forums, importance of non-emotional view e.g. talking to strangers?

m. How do you make decisions about whether to change medications or add in other treatments – what influences those decisions? Who decides?

4. Now for a bit about day to day life. Tell me about a typical day with your dog(s) – your routine, walks, medications etc.

a. Was that routine based on advice from your vet, your own experience, changed with time?

b. Does the arthritis cause problems with getting in and out of the house, car, and stairs.

c. Have you had to adapt the house to help [your dog] or help you cope?

d. Is that changing with time – how do you know you need to make a different change?

e. Other dogs and how manage OA vs. non-OA dog (if applicable), especially with exercise, limiting play etc?

f. Do you do things differently on good days versus bad days? What are the things which the dog finds easy/difficult now? How do you manage those?

g. Are there things which your dog used to enjoy doing which he/she now can’t do?

h. How has your dog coped with not being able to do the things they were doing before? Have you added anything to replace those activities?

i. Things which affect [the dog] positively or negatively – exercise, damp?

j. Changes in [the dog] other than physical health – attitude, mentation, play etc? Have interactions with other dogs changed at all?

k. [If appropriate, you have another younger dog – did you worry about getting a second dog when this one had arthritis? What difference has that made to your workload?]

5. Now on to your relationship with your dog and how you are coping with them having OA. Do you think your relationship with (dog’s name) has changed since he/she developed arthritis?

a. A study looking at dogs with other chronic diseases said people worry much more about their pets when they get ill and feel more like a carer – do you agree?

b. What aspects worry you the most?

c. To what extent does having a dog with OA interfere with your normal day to day life? Problems with lifting etc?

d. Has the fact your dog has arthritis had effects on any other members of your family? Children, holidays, visitors coming round, days out?

e. Is it harder to put the dog in kennels or get help with a dog sitter if needed because of the arthritis?

f. Has it affected your social contact e.g. contact with other dog walkers?

g. Finance if appropriate? Insured? Does it pay?

6. Now on to something a bit different. Do think the term quality of life is relevant to dogs? What do you think it means – is it something you think about?

a. Have you discussed quality of life with your vet?

b. How do you think they judge it? How do you judge it?

c. Do you think you judge your dog’s quality of life in a different way to your vet?

d. To have a good quality of life, what do you think your dog needs?

e. Has this changed with age?

7. Can we talk more now about decision making in the past and the future- others have said this is one of the hardest things to know how to assess their dog as it gets older – do you agree?

a. Others have said they really worry about others worrying about decision making as their dog gets older – do you agree?

b. Dog as a semi-public figure – thoughts about what other dog walkers might think of walking an older stiff dog?

c. Involvement of family and friends – are any decisions you make your own or do you have to involve what others would think too e.g. friends and family?

d. Decision making about new treatments, end of life thoughts – how to decide when enough is enough?

e. Difference if first dog/dog which has been through a lot with them?

f. Feelings about this?

g. Where will get advice from – vet, family etc – importance of an objective view?

h. Whether having OA changes thoughts on other diseases – would it make more or less likely to see vet advice about e.g. lumps, dental disease? “Ignore stuff because you don’t want to know?”

8. Finally would you have any messages for other dog owners of vets – top tips, areas for future research etc?

a. Owners

b. Vets

c. Questions for any HCPs – areas of research?

d. Resources which they would find useful? Diagnostic, monitoring, general information? Dog forums, arthritis nurse clinic, more advice on what to monitor

9. Is there anything else you would like to talk about which we haven’t covered?
